# The impact of pediatric intensivists on the management of pediatric diabetic ketoacidosis in pediatric intensive care units

**DOI:** 10.1186/s12887-023-04398-z

**Published:** 2023-11-13

**Authors:** Ah Young Choi, Esther Park

**Affiliations:** 1 Department of Pediatrics, Chungnam National University Hospital, Daejeon, Republic of Korea; 2 Department of Pediatrics, Jeonbuk National University Children’s Hospital, Jeonju, Republic of Korea; 3 Research Institute of Clinical Medicine of Jeonbuk National University-Biomedical Research Institute of Jeonbuk National University Hospital, Jeonju, Republic of Korea

**Keywords:** Diabetic ketoacidosis, DKA, Pediatric intensivist, Pediatric intensive care unit, PICU

## Abstract

**Introduction:**

The impact of pediatric intensivists on managing pediatric patients with diabetic ketoacidosis (DKA) remains unknown. This study aimed to evaluate the impact of pediatric intensivists on outcomes in pediatric intensive care units (PICUs).

**Methods:**

This was a two-institution retrospective study of patients with DKA admitted to the PICU between 2012 and 2023. Pediatric patients (< 19 years of age) were included if they met the moderate to severe DKA criteria on PICU admission. The patients were subsequently divided into two groups based on the presence or absence of a pediatric intensivist. The primary outcome was the PICU length of stay (LOS). Secondary outcomes were adverse events during DKA treatment, hospital LOS, and mortality.

**Results:**

Fifty-two patients admitted to the PICU with a median age of 13.00 years (range, 0–18 years) were included; 32 (61.54%) were female. Patients managed by pediatric intensivists had significantly shorter PICU LOS (2.52 vs. 3.69 days, p < 0.05). Also, adverse events during DKA treatment were significantly decreased in the high-intensity group compared to the low-intensity group (12.50% vs. 50.00%, p < 0.05).

**Conclusions:**

High-intensity ICU staffing was associated with shorter PICU LOS and lower adverse events in pediatric patients with DKA. Our results suggest that dedicated pediatric intensivists can improve outcomes of critically ill pediatric patients with DKA.

## Introduction

Diabetic ketoacidosis (DKA) is a life-threatening disease in pediatric patients with diabetes mellitus. Management for DKA should be performed in a center experienced in DKA treatment, and immediate treatment in a pediatric intensive care unit (PICU) should be considered for children with severe DKA [1].

Intensivists are crucial in managing intensive care units (ICUs) and providing high-quality intensive care [2]. High-intensity ICU staffing (either mandatory intensivist consultation or closed ICU [all treatment directed by intensivist]) has been reported to reduce mortality, ICU length of stay (LOS), and mechanical ventilation duration compared to low-intensity ICU staffing (no intensivist or elective intensivist consultation) [3, 4]. However, there is a lack of studies on the impact of pediatric intensivists in the PICU. The impact of pediatric intensivists on managing pediatric patients with DKA has yet to be studied. DKA treatment is protocolized in many hospitals, and there is little variability in treating these patients, making it an ideal group to evaluate the impact of pediatric intensivists. We aim to evaluate the impact of pediatric high-intensity ICU staffing on outcomes in PICUs.

## Research design and methods

### Study design

Patients with moderate to severe DKA, aged < 19 years, admitted to the ICU from February 2012 to March 2023 were included. Data were collected retrospectively from two tertiary academic medical centers (Jeonbuk National University Children’s Hospital and Chungnam National University Hospital). The patients were divided into the high-intensity group, in which the patients were treated in PICUs staffed with ICU board-certified intensivists, and the low-intensity group, in which the patients were treated in PICUs without board-certified intensivists.

### Intensive care unit setting

#### Hospital A

The PICU at Jeonbuk National University Children’s Hospital opened in March 2013 with 10 beds, including two isolation rooms for medical and surgical patients. The nurse-to-patient ratio is approximately < 1:2. In March 2021, the PICU was converted to a high-intensity staffing model managed by a pediatric intensivist. The pediatric intensivist is a pediatrician board-certified in pediatric and critical care medicine. The pediatric intensivist and one pediatric resident are in the PICU during the daytime (8:00 AM – 6:00 PM) on weekdays. The intensivist does not stay in the hospital at night but is available to guide patient care by phone and to return to the PICU within 20 to 30 min if necessary.

#### Hospital B

The PICU opened in 2020 with 5 isolation rooms for medical and surgical patients, including cardiac surgery. Before 2020, pediatric patients used 3 beds in the adult ICU. The nurse-to-patient ratio was maintained at < 1:2. Since 2017, one pediatric intensivist has worked in the ICU (closed ICU) and is a pediatrician board-certified in pediatric cardiology and critical care medicine. The pediatric intensivist and one resident are in the PICU during the daytime.

Before the implementation of high-intensity ICU staffing, a resident acted as the primary decision-maker for managing patients with DKA in both hospital ICUs. They were supervised by a pediatric endocrinologist for DKA management, and there was no option for consultation with an intensivist.

All patients with DKA were managed according to the published management guidelines during all periods in both hospitals.

### Definitions of diagnostic criteria and outcome measures

The DKA diagnosis was based on the guidelines of the International Society for Pediatric and Adolescent Diabetes 2022. The following should be present: hyperglycemia (random glucose level > 200 mg/dL), venous pH < 7.3 or serum bicarbonate < 18 mmol/L, and ketonuria (≥ 2+) [1]. DKA severity was categorized based on the degree of acidosis at admission: mild (pH < 7.3 or bicarbonate < 18 mmol/L), moderate (pH < 7.2 or bicarbonate < 10 mmol/L), or severe (pH < 7.1 or bicarbonate < 5 mmol/L) [1]. We only included patients with moderate to severe DKA in this study.

Clinical characteristics included sex, age, body mass index (BMI), height, body weight, type of diabetes, new-onset diabetes, Glasgow coma scale (GCS) at admission, insurance level, and precipitating factors. BMI, height, and body weight were converted into standard deviation scores (SDS) using the 2017 Korean National Growth Charts [5]. After reviewing the chart, current medication, and laboratory data, such as antibody and C-peptide, the type of diabetes (type 1 or 2) was identified. The insurance levels that applied to medical expense coverage were National Health Insurance and Medical Aid.

Laboratory data were collected with initial blood gas analysis, including pH, bicarbonate, base excess, anion gap, level of total CO2, and lactate level. In addition, random glucose, serum electrolyte, blood urea nitrogen, creatinine, HbA1c, serum C-peptide, urine ketone, white blood cell count, hematocrit, platelet count, and C-reactive protein levels were collected. The estimated glomerular filtration rate was calculated using the updated Schwartz Eq. [6].

The primary outcome was PICU LOS. The secondary outcomes were the rate of adverse events during DKA management in PICU, complications of DKA, hospital LOS, time to DKA resolution, and hospital mortality.

Possible adverse events during DKA management include hypoglycemia (blood glucose < 70 mg/dL), severe hypokalemia (serum potassium < 2.5 mEq/L), moderate to severe hypophosphatemia (serum phosphate < 2 mg/dL), fatal cardiac arrhythmias, and cerebral edema during the ICU stay. Possible complications of DKA include bloodstream infection confirmed by blood culture, shock requiring inotropic support, acute kidney injury requiring renal replacement therapy, and coagulopathic complications (e.g., deep vein thrombosis, cerebral venous thrombosis, stroke). The time to DKA resolution was determined by measuring the time between the patient’s arrival in the emergency department and the resolution of ketoacidosis, defined based on the following criteria from the American Diabetes Association Consensus Statement on Hyperglycemic Crises — plasma glucose < 200 mg/dL and 2 of the following: plasma bicarbonate ≥ 15mEq/L, venous pH > 7.3, and anion gap ≤ 12 mEq/L[7].

### Statistical analysis

The statistical analyses were performed using R Studio version 2022.07.02 (Build 576). To compare the groups, we used the Mann-Whitney U test for continuous variables and Fisher’s exact test for categorical variables. The data were presented as median and interquartile range (IQR) for continuous variables and as total number and percentage for categorical variables. A P value < 0.05 was considered statistically significant.

## Results

Of the 52 patients admitted to the ICU with moderate to severe DKA, 16 patients received treatment in the high-intensity ICU setting (high-intensity group). In comparison, 36 patients were treated in the low-intensity ICU (low-intensity group). The baseline characteristics of both groups are presented in Table 1. The overall median age was 13.00 (9.00, 15.00) years, and 32 (61.54%) patients were female. The median BMI was − 0.40 (-1.56, 1.01) SDS. There were 41 patients with type 1 diabetes and 11 with type 2 diabetes. Thirty-eight patients were newly diagnosed with diabetes, of which 30 were diagnosed with type 1 diabetes, and 8 were diagnosed with type 2 diabetes. At admission, median HbA1c was 12.95 (11.17, 13.77) %, and random glucose level was 486.00 (377.25, 604.25) mg/dL. The mean GCS at admission was 15.00 (12.00, 15.00), and 2 (3.85%) patients were in a coma state (less than 8 points on the GCS) at admission. The high-intensity group showed a decreased level of pH (7.04 vs. 7.12, p = 0.028). The anion gap, serum osmolality, and hematocrit increased in the high-intensity group (anion gap, 27.45 vs. 20.95 ml/dL, p = 0.011; serum osmolality, 321.50 vs. 315.50 mOsm/Kg, p = 0.038; hematocrit, 47.70 vs. 41.75%, p = 0.002).

**Table 1:** Baseline characteristics of patients ICU-hospitalized with diabetic ketoacidosis

Clinical characteristics	Overall (n = 52)		Low-intensity ICU (n = 36)		High-intensity ICU (n = 16)		*P*-value
Sex, female (%)	32 (61.54)		24 (66.67)		8 (50.00)		0.356
Age, years	13.00 (9.00, 15.00)		13.00 (8.50, 15.00)		12.50 (10.00, 14.25)		0.842
Height, SDS^a^	0.22 (-0.47, 0.79)		0.11 (-0.47, 0.77)		0.28 (-0.22, 1.29)		0.420
Weight, SDS	-0.61 (-1.81, 0.84)		-0.63 (-1.36, 0.59)		0.50 (-0.97, 2.11)		0.127
BMI, SDS^b^	-0.40 (-1.56, 1.01)		-0.40 (-1.66, 0.52)		-0.29 (-1.48, 1.98)		0.393
Diagnosis							0.073
Type 1 diabetes (%)	41 (78.85)		31 (86.11)		10 (62.50)		
Type 2 diabetes (%)	11 (21.15)		5 (13.89)		6 (37.50)		
Severity of DKA							0.068
Moderate (%)	20 (38.46)		17 (47.22)		3 (18.75)		
Severe (%)	32 (61.54)		19 (52.78)		13 (81.25)		
New-onset diabetes (%)	38 (73.08)		28 (77.78)		10 (62.5)		0.316
Weight loss, %	8.05 (4.75, 12.98)		7.95 (4.55, 12.22)		12.25 (5.62, 15.48)		0.254
Glasgow coma scale	15.00 (12.00, 15.00)		15.00 (12.00, 15.00)		15.00 (12.75, 15.00)		0.746
Coma state (%)	2 (3.85)		2 (5.56)		0 (0.00)		0.642
Insurance							0.562
Medical aid (%)	9 (17.31)		5 (13.89)		4 (25.00)		
National health insurance (%)	43 (82.69)		31 (86.11)		12 (75.00)		
Precipitating factors							0.133
Infection	16 (30.77)		14 (38.89)		2 (12.50)		
Non-compliance to treatment	6 (11.54)		2 (5.56)		4 (25.00)		
Unknown causes	10 (19.23)		7 (19.44)		3 (18.8)		
Indiscretion with sugar	6 (11.54)		3 (8.33)		3 (18.75)		
Puberty	14 (26.92)		10 (27.78)		4 (25.00)		
Initial laboratory data							
pH	7.08 (6.96, 7.16)		7.12 (7.01, 7.19)		7.04 (6.93, 7.08)		0.028
pCO2, mmHg	14.95 (12.15, 20.27)		14.95 (12.00, 20.27)		14.95 (14.00, 18.80)		0.758
Bicarbonate, mmol/L	4.25 (3.00, 6.43)		4.85 (3.08, 6.70)		4.05 (2.92, 4.80)		0.171
Random glucose, mg/dL	486.00 (377.25, 604.25)		486.00 (377.25, 640.75)		493.50 (379.50, 541.00)		0.634
Serum osmolality, mOsm/Kg^c^	317.50 (308.75, 323.00)		315.50 (307.25, 319.50)		321.50 (317.25, 337.50)		0.038
HbA1c, %	12.95 (11.17, 13.77)		12.95 (11.17, 13.70)		13.25 (11.25, 14.05)		0.670
Corrected sodium, mmol/L	138.80 (136.20, 142.33)		137.80 (136.15, 141.05)		140.20 (138.05, 146.93)		0.106
Potassium, mmol/L	4.15 (3.68, 4.60)		4.10 (3.70, 4.60)		4.35 (3.58, 4.85)		0.781
Anion gap, ml/dL	21.90 (19.38, 28.08)		20.95 (18.17, 24.77)		27.45 (20.25, 30.00)		0.011
Base excess, mmol/L	-25.00 (-27.55, -21.85)		-24.15 (-27.75, -20.98)		-26.05 (-27.30, -24.20)		0.211
Lactate, mmol/L	1.50 (1.00, 2.25)		1.45 (1.00, 2.00)		1.75 (0.98, 3.15)		0.388
BUN, mg/dL	16.00 (11.75, 20.77)		16.05 (11.75, 20.70)		16.00 (11.82, 21.02)		0.827
Creatinine, mg/dL	0.94 (0.70, 1.16)		0.91 (0.65, 1.13)		1.03 (0.82, 1.20)		0.134
eGFR, mL/min/1.73m^2,d^	66.47 (54.20, 75.07)		67.11 (56.06, 78.58)		56.69 (46.95, 72.62)		0.153
C-peptide serum, ng/mL	0.41 (0.19, 0.83)		0.41 (0.29, 0.64)		0.41 (0.16, 1.66)		0.970
Ketone, urine dipstick	4.00 (3.75, 4.00)		4.00 (4.00, 4.00)		4 (3.00, 4.00)		0.144
WBC, 10^3^/ul	19.01 (10.31, 28.84)		20.04 (10.42, 29.91)		14.70 (10.17, 24.25)		0.500
Hematocrit, %	43.15 (40.90, 47.65)		41.75 (38.77, 45.83)		47.70 (42.85, 50.80)		0.002
CRP, mg/L	1.72 (0.28, 9.14)		1.88 (0.32, 6.90)		0.80 (0.19, 11.70)		0.648

Data are presented as the median and interquartile range or number of patients (%)

ICU, intensive care unit; BMI, body mass index; SDS, standard deviation score; DKA, diabetic ketoacidosis; BUN, blood urea nitrogen; eGFR, estimated glomerular filtration rate; WBC, white blood cells; CRP, C-reactive protein

The items marked with small letters represent the total number of items as follows: ^a^Height (n = 46; low-intensity ICU, n = 30; high-intensity ICU, n = 16), ^b^BMI (n = 46; low-intensity ICU, n = 30; high-intensity ICU, n = 16), ^c^Serum osmolality (n = 32; low-intensity ICU, n = 22; high-intensity ICU, n = 10) and ^d^eGFR (n = 46; low-intensity ICU, n = 30; high-intensity ICU, n = 16)

The adverse events during treatment for DKA, including hypoglycemia, hypokalemia, and hypophosphatemia, were seen in 20 patients (38.46%) during the ICU stay (Table 2). Adverse events occurred less frequently in the high-intensity ICU group than in the low-intensity ICU group (2 patients [12.50%] vs. 18 patients [50.0%], p = 0.014). No cardiac arrhythmia or cerebral edema was reported in our study.

**Table 2:** The adverse events during treatment for diabetic ketoacidosis in PICU.

	Overall (n = 52)	Low-intensity ICU (n = 36)	High-intensity ICU (n = 16)	*P*-value
Total, n (%)	20 (38.46)	18 (50.00)	2 (12.50)	0.014
Hypoglycemia, n (%)	16 (30.77)	14 (38.89)	2 (12.50)	0.102
Hypokalemia, n (%)	5 (9.62)	5 (13.89)	0 (0.0)	0.308
Hypophosphatemia, n (%)	4 (7.69)	4 (11.11)	0 (0.0)	0.299

Data are presented as the number of patients (%)

PICU, pediatric intensive care unit

The PICU LOS was significantly shorter in the high-intensity group than in the low-intensity group (2.52 [1.19, 3.86] vs. 3.69 [2.55, 4.91] days, p = 0.037) (Table 3). There was no significant difference in hospital LOS between the two groups (13.00 [10.75, 16.25] vs. 12.00 [10.75, 16.25] days, p = 0.905). Time to DKA resolution was significantly longer in the high-intensity group than in the low-intensity group (33.01 [25.13, 44.81] vs. 19.87 [12.52, 30.96] hours, p = 0.007).

**Table 3:** Management and outcomes of DKA.

		Overall (n = 52)	Low-intensity ICU (n = 36)	High-intensity ICU (n = 16)	*P*-value
ICU length of stay (days)		3.28 (2.28, 4.60)	3.69 (2.55, 4.91)	2.52 (1.19, 3.86)	0.037
Hospital length of stay (days)		12.00 (10.75, 16.25)	12.00 (10.75, 16.25)	13.00 (10.75, 16.25)	0.905
Duration of insulin infusion (hours)		35.53 (25.67, 55.10)	34.16 (24.25, 48.50)	41.38 (28.02, 63.52)	0.388
Time to DKA resolution (hours)		22.88 (14.40, 35.56)	19.87 (12.52, 30.96)	33.01 (25.13, 44.81)	0.007
Mechanical ventilation, n (%)		2 (3.85)	1 (2.78)	1 (6.25)	0.525
Complication of DKA, n (%)		1 (1.92)	1 (2.78)	0 (0.0)	1.000

Data are presented as the median and interquartile range or number of patients (%)

DKA, diabetic ketoacidosis; ICU, intensive care unit

Only 2 patients required mechanical ventilation due to loss of consciousness and seizure, and none required renal replacement therapy, vasopressors, or inotropic treatment. There were no deaths or blood culture-proven sepsis; one patient developed ischemic stroke in the low-intensity ICU group.

## Discussion

This is the first study to explore associations between the presence of pediatric intensivists and PICU outcomes for children with DKA. Our results showed that a dedicated pediatric intensivist contributed to reducing ICU LOS in pediatric DKA patients and reducing adverse events that could occur during treatments for DKA.

When ICU staffing transitions from low-intensity to high-intensity, ICU outcomes are significantly improved [3, 4]. However, the finding is inconsistent across PICU studies. Goh et al. demonstrated that pediatric intensivists were associated with improved mortality rates and a significant reduction of PICU LOS in a developing country [8]. Kesici et al. showed that dedicated pediatric intensivists were associated with improved clinical outcomes, including mortality rate, mechanical ventilation duration, and ICU LOS, in a middle-income country [9]. Nishisaki et al. found that implementing a 24-hour in-hospital pediatric intensivist was associated with a shorter ICU LOS and duration of mechanical ventilation [10]. Gupta et al. showed that 24-hour intensivist presence was associated with improved survival, shorter ICU LOS, and shorter duration of mechanical ventilation [11].

In contrast, Pollack et al. and Kim YH et al. have shown that pediatric intensivists do not significantly affect PICU LOS [12, 13]. The mixed results in several studies are due to other organizational factors such as primary care practices, multidisciplinary team support, nurse-led quality, and type of ICU, and all of these factors can affect PICU outcomes [14]. The existence of intensivists is not the sole factor influencing ICU outcomes [2]. There are published guidelines for DKA, and most experienced hospitals use protocolized treatment in patients with DKA. The clinical outcome of DKA treatment is thought to be relatively less affected by the factors described above compared to other diseases that require ICU admission, so we evaluated the impacts of a dedicated pediatric intensivist in pediatric patients with DKA requiring ICU admission.

DKA has many complications, including mortality, permanent severe neurologic sequelae, acute kidney injury, hypoglycemia, electrolyte imbalance, and coagulopathic complication [1]. Several studies evaluated the factors affecting PICU outcomes in DKA management [15–17]. Slain et al. evaluated the relationship between the household income of patients and PICU LOS in children with DKA [15]. Low income was not associated with longer PICU LOS [15]. Children with prolonged acidosis (lasting more than 24 h) had longer PICU LOS (1.86 ± 0.86 vs. 1.42 ± 0.63 days, p < 0.05) [17]. Shenoy et al. also showed longer ICU LOS in the prolonged acidosis group than in the control group (4.22 ± 2.8 vs. 2.86 ± 1.56 days, p = 0.038) [16]. There has been no study on the impact of intensivists on patients admitted to PICUs for DKA treatment. Our study included patients with moderate to severe DKA admitted to the PICUs. The presence of an intensivist decreased the rate of adverse events and the ICU LOS. Despite the same treatment guidelines for DKA, the reduction in adverse events is thought to result from frequent bedside education for residents and nurses by the intensivist and the intensivists’ direct personal involvement in clinical care. Reducing the incidence of treatment complications and medical errors improves treatment quality and greatly helps patient safety. In addition, intensivists can also reduce ICU LOS by making appropriate ICU discharge decisions. This may result in more efficient use of medical resources and lowered medical costs.

Our study has some limitations. The sample size of 52 patients is relatively small, which may limit the generalizability of the findings. Future studies with larger sample sizes across multiple institutions are needed to corroborate these results. In addition, the retrospective design in two hospitals is the most significant limitation. It could potentially introduce bias or limit the applicability of the findings to other settings. Expanding the scope to include multiple institutions with varied operational and staffing models could provide more comprehensive insights.

## Conclusion

High-intensity ICU staffing involving dedicated pediatric intensivists was associated with reductions in PICU LOS and adverse events of DKA treatment in patients with DKA. Our results suggest that pediatric intensivists can improve outcomes of critically ill pediatric patients with DKA.

## Data Availability

The datasets analyzed in this study are available from the corresponding author upon reasonable request.

## Abbreviations

DKA: Diabetic ketoacidosis
PICU: Pediatric intensive care unit
LOS: Length of stay
GCS: Glasgow coma scale
